# Impact of data compositionality on the detection of microbiota responses

**DOI:** 10.1080/19490976.2025.2590841

**Published:** 2025-12-02

**Authors:** Brandon Hickman, Katri Korpela

**Affiliations:** a Human Microbiome Research Program, Faculty of Medicine, University of Helsinki, Helsinki, Finland; b Department of Bacteriology and Immunology, University of Helsinki, Helsinki, Finland

**Keywords:** Compositional data, next generation sequencing, false discovery rate

## Abstract

Next-generation sequencing (NGS) data usage is widespread, but its compositional nature poses challenges. We evaluated four normalization methods (relative abundance, CLR, TMM, DESeq2) for identifying true signals in compositional microbiota data using simulations. Two experiments were conducted: one with only increases in specific taxa, and a 1:1 increase/decrease in specific taxa. Simulated sequencing produced compositional data, which were normalized using the four methods. The study compared absolute abundance data and the normalized compositional data using variance explained and false discovery rates. All normalization methods showed decreased variance explained and increased false positives and negatives compared to absolute abundance data. CLR, TMM, and DESeq2 did not improve over relative abundance data and sometimes worsened false discovery rates. The study highlights that false positives and negatives are common in compositional NGS datasets, and current normalization methods do not consistently address these issues. Compositionality artefacts should be considered when interpreting NGS results and obtaining absolute abundances of features/taxa is recommended to distinguish biological signals from artefacts.

## Introduction

Microbiota data are typically generated via high-throughput sequencing, resulting in a dataset of read counts per taxon per sample. The taxa can be summarised at different levels, e.g., genera, species, operational taxonomic units (OTUs) or amplicon sequencing variants (ASV). These are the measured variables, i.e. the features in the data. Such data are inherently compositional in nature. Compositional data have a constant-sum constraint, meaning that all measured variables, or features in the data, sum to 100%. This constraint is imposed because the sequencing instrumentation has an upper limit on the number of reads, which introduces dependence between features and creates spurious correlations.

The number of reads per sample varies and does not carry biological meaning; therefore, the data must be normalized to account for variable read counts between samples. The number of reads per sample is useful only as a quality assessment and precision indicator. Consequently, the counts of each feature in the dataset are interpretable only relative to all other features in the sample.

From a biological perspective, the problem is that of interpretation. First, an increase in the relative abundance of one taxon, does not necessarily indicate an absolute increase in that taxon but could arise from a decrease in other taxa. It is impossible to distinguish true biological responses from compositional datasets, which can lead to misinterpretations. Second, compositional data ignore variation in total microbial abundance^1^,which influences the total abundance of microbial metabolites and,in the case of host-associated microbiomes,immunologically active components and is likely biologically relevant.^1^

From a statistical perspective, compositional data are problematic due to the dependence between features, i.e., the units that have been identified and measured, such as species or OTUs: a high relative proportion of one feature occurs at the expense of the other features. This violates the assumptions of many statistical analyses, such as ordination methods^2^ and co-abundance correlations,^3^^,^^4^ which assume independence between observations. Another issue is that the relative data are constrained between 0 and 1, and their distributions are not normal, often even after logarithmic transformation. Thus, statistical techniques that assume independence of data points and normal distributions are not applicable, although they are commonly used, leading to unreliable *p* values.

These statistical problems are typically addressed by applying data transformations. Several methods have been recommended for different operations^5-7^. The relative abundance (read count normalization, i.e., dividing the read counts of the features by the total read count of the sample) is the most common transformation, but is often considered insufficient. As a solution, many researchers recommend the centered log-ratio (CLR) transformation, which takes the log of the read counts in a sample divided by the geometric mean of the read counts in that sample, thus normalizing each feature relative to other features in the sample. CLR data are scale-irrelevant and represent the features’ abundances with respect to the mean. However, the transformed samples are still linearly dependent, as they have a determinant equal to zero, meaning that they have no inverse and a singular covariance matrix (when any row or column of a square matrix is the weighted sum of another). This is problematic for common statistical methods, as it cannot be used to solve systems of linear equations because there is no unique solution.^8^

Normalization methods have also been developed based on distributions of read count data. Using the trimmed mean of M values (TMM) in R package edgeR^9^ a reference sample is chosen, and the relative abundances of features are adjusted against those in the reference sample. TMM thus normalizes each feature in relation to the average feature-wise difference between the sample and a reference sample.^10^

Another, similar normalization method is DESeq2^11^. DESeq2 normalizes the feature counts by a size factor, determined by the median of the ratio of feature counts per sample against the geometric mean of the features in all samples; thus, DESeq2 uses a similar logic as TMM, but instead of a reference sample, each sample is compared to the average of all samples^11^. Both TMM and DESeq2 assume that most features are not differentially expressed so that differences from sample mean points become apparent.^10^

Using an *in-silico* experiment, we investigate the impact of data compositionality and the different data transformation methods on the observed results through the proportion of type I and type II errors and the degree of variance attributable to the treatment effect. We attempt to determine the optimal method, in terms of reliable output, for high-throughput sequencing data for microbiota studies.

## Methods

To test the effectiveness of data transformations on sequenced data, we created a simulated dataset of the absolute abundance of 100 features (taxa) for a total of 20 subjects. This roughly resembles a typical gut bacteriome data set at genus or family level. At this level, data sparsity is typically not a problem, since most humans harbor largely the same bacterial taxa at the genus or higher taxonomic levels. Because data sparsity is a separate problem, independent of compositionality, we did not address sparsity in this study.

**Figure 1.**
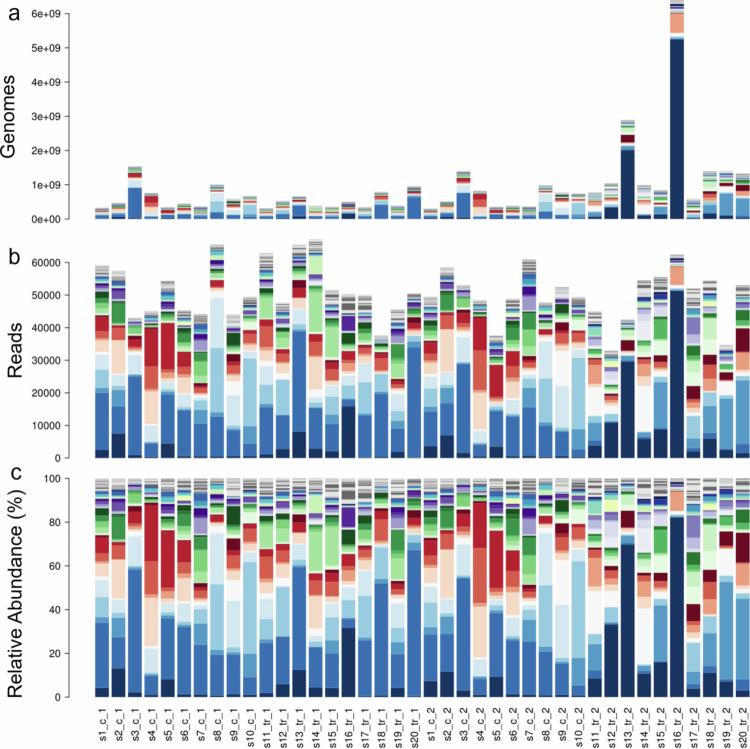
Microbial profiles for sample populations. a) the absolute abundance profiles for the 20-sample population for experiments 1 and 2, control “c” and treatment “t” groups. b) the sequenced data with a read count mean of 50000 and a standard deviation of 10000 reads per sample. c) the relative abundance transformation of the data.

A single subject’s microbial profile was initially created by calculating the exponential of 100 random numbers from a normal distribution with a mean of 12.5 and a standard deviation of 2.5 to obtain the absolute abundances of 100 taxa (copies of genomes). A mean of 12.5 was chosen as the total abundance of bacteria in the gut is on average 12.5 logs (per ml of feces)^12^^,^^13^. The standard deviation of 2.5 was empirically chosen as it resulted in a realistic microbiota profile with a few dominant taxa and many low-abundance taxa (Figure 1). Each additional subject was created as a variant of the first by multiplying the first subject’s taxon abundance by a random number from a normal distribution with a mean of 0 and a standard deviation of 0.5. This ensures that the taxa had similar abundances and thus are correlated across different samples, as in real microbiota datasets, rather than being randomly distributed or simulated taxon-by-taxon, which has been shown to be important in microbiota simulation experiments.^14^ Using this approach, a correlation structure between features, present in real microbial communities, was ensured.

The subjects were evenly distributed into a control (“c”, *N* = 10) and a treatment group (“t”, *N* = 10), and for each group, a postintervention profile was created based on the individual baseline profile (Figure 1a). Two experiments were run: 1) unidirectional, where all microbial responses to the treatment were positive, and 2) bidirectional, where both positive and negative responses at a 1:1 ratio were included. In addition to the treatment effect (for subjects in the treatment group), a random 25% change between time points for each taxon was implemented for all subjects. Thirty scenarios were created for testing the impact of effect size (magnitude of the individual taxon responses) and the proportion of taxa responding. The tested effect sizes were 0.1x, 0.5x, 1x, 5x, and 10x, with 6 different proportions of responding microbes at the given magnitude (1%, 5%, 10%, 25%, 50%, and 75%). Absolute abundance data underwent simulated 16S rRNA amplicon sequencing, with a read count mean of 50000 and a standard deviation of 10000 reads per sample (Figure 1b). The sequenced simulated data were then transformed by four methods: relative abundance (Figure 1c), CLR^7^,DESeq2^15^ and TMM using edgeR library.^16^

For each experiment, we assessed the impact of data transformation by calculating the false discovery rate (FDR) (false positive: FP; false negative: FN). To identify significantly responding taxa, we used the Kruskal‒Wallis rank sum test with an FDR-corrected *p* value (Benjamini‒Hochberg correction) less than 0.1 as the significance cutoff. We chose a nonparametric test that is not dependent on data distributions to be able to use the same test for all data transformations. The observed false positives and negatives of each dataset were then compared to the prevalence of errors in the non-transformed absolute abundance data. We compared the coefficient of determination (R^2^) to identify the observed variance explained by the treatment by performing a permutation multivariate ANOVA using the adonis2 function from the vegan package^17^ in R (v. 4.3.0). Three distance matrices (log-Pearson distance, Bray‒Curtis distance, and Aitchison distance [Euclidean distance calculated from CLR data]) were tested on the transformed compositional datasets. Each experiment was simulated 100 times, and we analyzed the mean variance explained and the mean FDR over the 100 iterations.

## Results

### The false discovery rate in absolute abundance data

**Figure 2.**
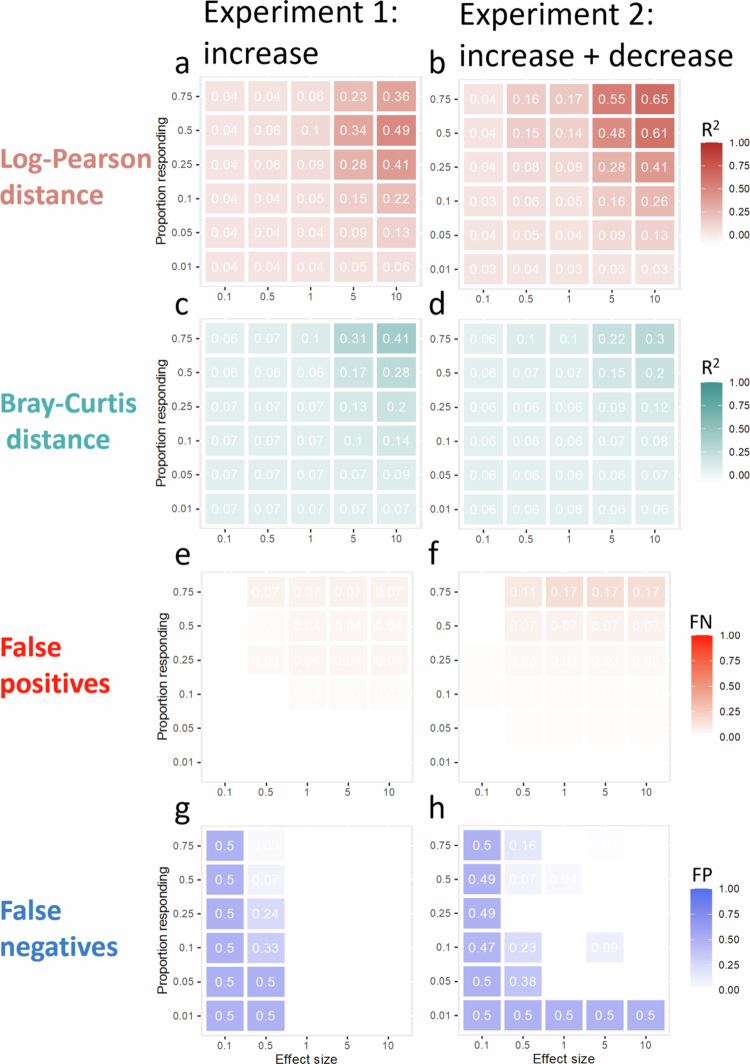
Total variance and false discovery rates in absolute abundance data. a) and b) the total variance explained for experiments 1 and 2 using log‒Pearson distance shown in dark red, and (c and d) the Bray‒Curtis distances shown in turquoise. e) and f) the false positive rates, shown in bright red, are shown for experiments 1 and 2. g) and h) the false negatives, shown in blue, for experiments 1 and 2. Each panel plots the effect size versus the proportion of responding.

In the simulated *in silico* absolute abundance microbiota data of 20 subjects at baseline and post-treatment, the variance explained (R^2^) by the treatment (multivariate ANOVA) increased with both the effect size and the proportion of taxa that responded (Figure 2a-d). The estimated R^2^ was generally greater when the log‒Pearson distance (1 - correlation) was used than when the Bray‒Curtis distance was used, especially in the second experiment (Figure 2b,d).

The false discovery rates at the FDR-corrected *p* value < 0.1 cutoff for the absolute abundance data (Figure 2e−h, Table 1) show that FPs are a minor problem, increasing to 17% only in the second scenario at a 75% response rate. Conversely, FNs are problematic when either the response rate or effect size is low.

**Table 1.** The maximum and mean false positives (FP) and false negative (FN) obtained from all scenarios. The FP and FN were calculated for the absolute abundance data and each of the data transformations: Relative abundance, CLR, DESeq2, and TMM. For the transformations we show the mean FP and mean FN fold changes in relation to the absolute abundance data.

	Maximum FP	Mean FP	Mean FP fold change vs absolute abundance	Maximum FN	Mean FN	Mean FP fold change vs absolute abundance
Experiment 1						
Absolute abundance	0.070	0.025	—	0.500	0.157	—
Relative abundance	0.500	0.145	5.800	0.500	0.256	1.631
CLR	0.480	0.166	6.640	0.500	0.365	2.325
DESeq2	0.500	0.152	6.080	0.500	0.278	1.771
TMM	0.500	0.115	4.600	0.500	0.305	1.943
Experiment 2						
Absolute abundance	0.170	0.037	—	0.500	0.198	—
Relative abundance	0.480	0.109	2.946	0.500	0.281	1.419
CLR	0.440	0.072	1.946	0.500	0.328	1.657
DESeq2	0.500	0.046	1.243	0.500	0.279	1.409
TMM	0.500	0.110	2.973	0.500	0.272	1.374

### False discovery rates in compositional data

**Figure 3.**
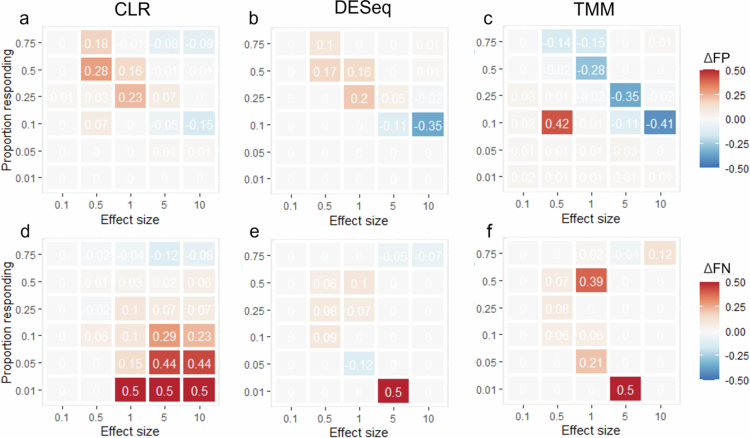
Differences in false discovery rate in comparison to relative abundance for experiment 1. The difference in false positives (∆FP) for CLR (a), DESeq2 (b), and TMM (c) and false negatives (∆FN) for CLR (d), DESeq2 (e), and TMM (f) in comparison to relative abundance for effect size and proportion responding to experiment 1. Each panel has also had the FP/FN for the absolute abundance removed to account for the inherent error in FDR.

**Figure 4.**
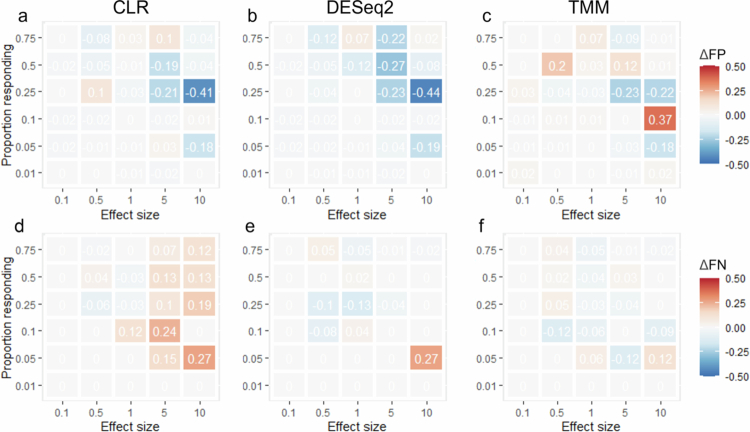
Differences in false discovery rate in comparison to relative abundance for experiment 2. The difference in false positives (∆FP) for a) CLR, b) DESeq2, and c) TMM and false negatives (∆FN) for d) CLR, e) DESeq2, and f) TMM in comparison to relative abundance for effect size and proportion responding for experiment 2. Each panel has also had the FP/FN for the absolute abundance removed to account for the inherent degree in FDR.

To determine the degree of false discoveries in the compositional datasets, we assessed the rates of FP and FN for four types of commonly used data transformation methods: relative abundance, CLR, DESeq2, and TMM. The rate of FPs increased by 5–6-fold in the compositional datasets compared with the absolute abundance data in experiment 1 and by 1–3-fold in experiment 2 (Table 1). The rate of FNs increased approximately 1- to 2-fold in both experiments (Table 1). Comparing the differences between relative abundance (read count normalization) and the other three transformations for experiment 1 (Figure 3) and experiment 2 (Figure 4), there was no clear improvement. For experiments that had only an increasing effect on the microbiota (experiment 1), both CLR and DESeq2 (Figure 3a,b) showed an overall increase in the degree of FP. TMM (Figure 3c), while resulting in some cases of lower FPs, also showed increased FPs at lower effect sizes and proportional responses. Compared to relative abundance data, FNs (Figure 3d−f) were more prevalent in the CLR, TMM, and DESeq2 datasets. With experiment 2 (1:1 increase-decrease) (Figure 4a and b), CLR and DESeq2 showed a general decrease in FP. TMM, however, had several instances of increased FP (Figure 4c). CLR increased the rate of FNs, whereas the other transformations had small and mixed effects on the FN rate (Figure 4d−f).

**Figure 5.**
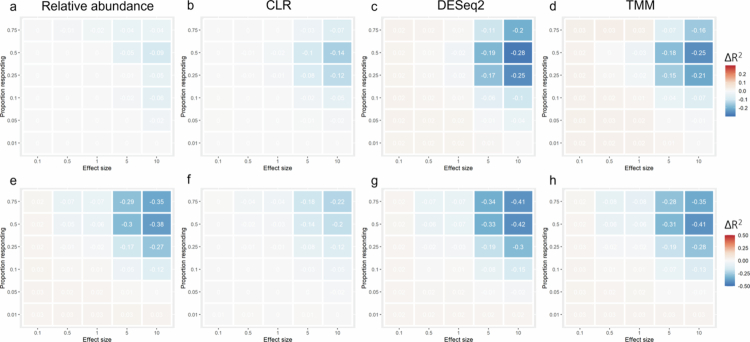
Differences in total variance explained for distances compared to the log-Pearson distance for each data transformation method. The total variance explained, R^2^, in experiment 1 when taking the difference of between Bray‒Curtis (or in case of the CLR, Euclidean distance) and the log-Pearson for the a) relative abundance, b) CLR, c) DESeq2, and d) TMM, and in experiment 2 for the e) relative abundance, f) CLR, g) DESeq2, and h) TMM.

The differences in R^2^ based on either Bray‒Curtis distance for relative abundance, DESeq2, and TMM, or Euclidean distance for the CLR (which equates to the Aitchison distance) compared with the log‒Pearson distance are shown in Figure 5 for both experiments. Overall, the generally recommended Bray‒Curtis and Aitchison distances result in an underestimation of the amount of variance explained in comparison with the log-correlation distance for all data transformations and both experiments. The largest differences were observed for DESeq2 and TMM in experiment 1 (Figure 5c,d) and across the board in experiment 2 (Figure 5e-h).

## Discussion

Using a simulated microbiota intervention with 20 subjects sampled twice and 100 taxa measured, we explored the impact of common data transformations on the reliability of the observed results. These four methods in this study were chosen as they are widely used in the field of microbiome research and thus special attention should be given to understand the consequences of choosing one of these methods. Our results highlight the main compositionality artifacts and the increased rate of both false positives and false negatives and show that the often-recommended methods for dealing with compositional data fail to eliminate these artifacts. These results demonstrate that high-throughput sequencing studies suffer from high degrees of false discoveries and that currently used data transformations are not an effective solution to overcome this problem.

Certain types of microbiota-targeting treatments could be expected to have mainly unidirectional effects, such as prebiotic (expected to increase the abundance of bacteria capable of utilizing the substrate) or antibiotic (expected to decrease the abundance of sensitive bacteria). However, more complex treatments, such as dietary interventions, likely have both positive and negative effects. By performing two different kinds of experiments, one with only positive effects on the taxa and another with 1:1 positive and negative effects, at 35 different variations regarding the proportion of taxa responding and the effect size, we expect to have covered many different types of biological scenarios. Importantly, based on relative abundance data, such differences cannot be distinguished, because all treatments will appear to have bidirectional effects due to the compositionality of the data: it is not possible for relative abundances to only increase or decrease.

We created an absolute abundance dataset as the basis for understanding the true degree of FPs and FNs that are inherent in the data. As expected, using the FDR-correct *p*-value cut-off of 0.1, FP remained low throughout both experiments^18^ We discovered that for treatments that have large or widespread effects, FPs are more likely, and thus, the *p*-value cutoff should be strict, whereas for treatments with small effect sizes, FNs are more likely, and the *p*-value cutoff should be looser. The results indicate that it would be useful to determine one’s effect size, response rate and response type (unidirectional or bidirectional) to understand the FDRs and thus adjust the *p*-value cutoff accordingly. Owing to the nature of compositional data, this is inherently impossible, as any increase in the relative abundance of one taxon leads to a decrease in another, masking the real change.

All methods of relative data transformation fail to control FDR and underestimate the variance explained by the treatment. Regardless of the data transformation method, the FPs and FNs were several-fold greater when compared to the absolute abundance data. This is problematic, as it cannot be solved by adjusting the *p* value cutoff. Our results indicate that microbiota studies based on compositional data both report artifactual taxon associations and fail to identify many true associations. With FNs of up to 50%, a great deal of information is lost. The high FDR rate likely explains the varying effects observed between microbiota studies and the consequent difficulty in generalizing the results.

Our results suggest that directionality in the relative abundance of specific taxa in intervention studies may be misreported because of compositionality artifacts. As an example, the enrichment of *Ruminoccus* and *Prevotella* in plant-based (high-fiber) diets has been reported in several studies;^19–24^ however, a fiber intervention study using absolute abundances, obtained via the spike-in method, revealed a decrease in *Ruminococcus* with no increase in any taxa*^23^*. In addition, absolute abundance data revealed that the temporal development of the dominant taxa in the infant gut over time followed completely different patterns than observed using relative abundance data.^25^ One must look askance at microbiota studies reporting only relative abundances, as both directionality and effect sizes may be distorted due to the compositionality of the data.

Because of the high prevalence of FNs in relative data, studies utilizing absolute abundances can reveal unseen associations. Cesarean section-born children and those born to mothers receiving intrapartum antibiotics were shown to have a significant increase in the absolute abundance of streptococci, staphylococci, enterococci, and clostridia, which was not observed in relative data^25^. Time-scale analysis of compositional data is similarly flawed in that truly differentiating between increases and decreases is not possible, and inferences from the variability of bacterial relative abundance are prone to misinterpretation.^5^

In our study, all methods (CLR, TMM, DESeq2) underperformed compared with the use of simple relative abundance as read count normalization. This supports an earlier observation by Hawinkel, Mattiello^14^,which showed that no single method of normalization outperformed any other method and that all methods failed to control the FDR at a nominal value of 0.05. Comparisons between DESeq2 and TMM using transcriptomic data have shown that TMM performs poorly for genes with low expression levels and well with strongly expressed genes,thus maintaining reasonable FDR values. FDR values for both DESseq2 and TMM have even been reported to be greater than 20% for different sample sizes^26^^,^^27^ and generally have high FDR inflation^28–30^. Other studies have reported large variations in output when several commonly used data analysis tools are used, highlighting reproducibility concerns.^31^

Our results support previous studies suggesting that microbiota research should move towards absolute abundance measurements for improved accuracy and interpretability of the results. Linking absolute abundance quantification to high-throughput sequencing allows the use of traditional statistical testing and more straightforward interpretation of results, which are not available for compositional data. Absolute abundance is obtainable through several means, such as flow cytometry^18^,spike-in bacteria^32^, or^33^ total bacterial quantitative PCR,^34^ which can be used to translate the relative abundance profiles into absolute abundances^20^. While an enticing idea, predicting absolute abundances based on the relative abundance profiles is not a sustainable solution, since it does not allow for the relative abundances to be transformed into absolute abundances, and the predictions are potentially misleading.^33^

Notably, in addition to the compositionality problem, NGS data often suffer from zero inflation, where most features are detected only in a few samples, leading to sparse data. In microbiota data sets, this is especially common at low taxonomic resolutions (e.g. species level or in OTU tables), or with virome or fungome data, where different samples contain mostly different features. At low taxonomic levels zeros can represent up to 80−90% of the counts, but at higher taxanomic levels, zeros can be completely absent^35^, and thus the level of sparsity varies between data sets. Multiple methods have been tested to handle zero-inflated data,including adding pseudo counts,replacing zeros with small fractions,deep learning techniques,as well as the use of zero-inflated statistical models^36–38^. The simulated microbiota data in this study did not incorporate sparsity as it would create an added layer of complexity that is separate from the question of compositionality. While several distance metrics were considered in this study, many more exist such a phenotypic metrics(e.g. weighted/unweighted Unifrac) and could offer varying results^39^. Finally, a range of normalization methods exist beyond those discussed in this study and the benefits and disadvantages have been discussed elsewhere^40^. One such method is rarefying which is a library size sub-sampling without replacement^41^^,^^42^. Rarefying sets a minimum read count and then remove random samples from those above the minimum level. Studies have shown that rarefying lends itself towards a high false positive rate, and along with the loss of samples data, as well as alternative methods, it is generally recommended to avoid rarefying.^43^

## Conclusions

These data emphasize the importance of absolute abundance in terms of reducing false discovery rates and variance in high-throughput sequencing of microbial data. Using an *in silico* simulation of 20 subjects and two treatment types, we show that the data transformations commonly utilized in microbial studies are prone to false positives and negatives and underestimate the explainable variance in comparison to absolute abundances. Additionally, we compared how different distance metrics reduce the variance explained and propose the use of the log-Pearson distance as the optimal distance metric of those tested. This information can be used to improve data analysis and recover meaning and accurate results in microbial studies.

## Data Availability

The datasets generated during and/or analysed during the current study are available in the Microbial-Composition repository, [https://github.com/bhick001/Microbial-Composition].^44^
